# Distribución de *Aedes albopictus* en Ibagué: potencial riesgo de brotes de arbovirosis

**DOI:** 10.7705/biomedica.7010

**Published:** 2023-12-01

**Authors:** Cristian Camilo Canizales, Julio César Carranza, Gustavo Adolfo Vallejo, Daniel Alfonso Urrea

**Affiliations:** 1 Laboratorio de Investigaciones en Parasitología Tropical, Universidad del Tolima, Ibagué, Colombia Universidad del Tolima Universidad del Tolima Ibagué Colombia

**Keywords:** Aedes, arbovirus, control de vectores de las enfermedades, Colombia, Aedes, arboviruses, vector control of diseases, Colombia

## Abstract

**Introducción.:**

*Aedes albopictus* es un vector de arbovirus como dengue, Zika, chikungunya y fiebre amarilla. Los primeros reportes en el continente americano datan de 1985 y dada su capacidad de adaptación ecológica y fisiológica, se ha distribuido rápidamente en el territorio colombiano desde su primer reporte en 1998.

**Objetivo.:**

Determinar la distribución de *A. albopictus* en las comunas de Ibagué, Colombia.

**Materiales y métodos.:**

Los muestreos se realizaron entre mayo y noviembre de 2022 en zonas con abundante vegetación de las 13 comunas de Ibagué. Se emplearon aspiradores y redes entomológicas. Los mosquitos fueron transportados al Laboratorio de Investigaciones en Parasitología Tropical de la Universidad del Tolima para su determinación taxonómica.

**Resultados.:**

Se identificaron 708 ejemplares de *A. albopictus,* distribuidos en las comunas de Ibagué. La mayor abundancia del vector se presentó en las comunas 10, 11, 7, 8, 2 y 9. Las comunas 3, 4, 5, 6, 12 y 13 presentaron abundancias relativas cercanas al 3 %, y la comuna 1 tuvo una abundancia del 2 %.

**Conclusiones.:**

*Aedes albopictus* está distribuido en todas las comunas de Ibagué, probablemente su dispersión se ha visto favorecida por las condiciones ambientales y sociales de esta región. Se recomienda hacer seguimiento anual a las poblaciones de este vector y realizar una caracterización molecular de los arbovirus encontrados. Además, el conocer la distribución de este mosquito en la ciudad permitirá focalizar las estrategias de control entomológico y prevenir futuros brotes de arbovirosis.

## Abstract (en)

**Introduction.:**

*Aedes albopictus* is a vector for arboviruses, such as dengue, Zika, chikungunya, and yellow fever. The first *A. albopictus* reports on the American continent date back to 1985. It has spread rapidly throughout Colombia since its first report in 1998 due to its ecological and physiological adaptation capability.

**Objective.:**

To determine *A. albopictus* distribution in the 13 communes of Ibagué, Colombia.

**Materials and methods.:**

Samples were collected between May and November 2022 in the 13 communes of Ibagué. Vacuum sampling and sweep-netting entomological nets were used in areas with abundant vegetation. The mosquitoes were transported to the *Laboratorio de Investigaciones en Parasitología Tropical* at the *Universidad del Tolima* for taxonomic determination.

**Results.:**

We identified 708 *A. albopictus* specimens distributed throughout Ibague's 13 communes. The highest vector abundance occurred in communes 10, 11, 7, 8, 2, and 9; communes 3, 4, 5, 6, 12, and 13 had a relative abundance of around 3%, while commune 1 had 2% of relative abundance.

**Conclusions.:**

*Aedes albopictus* is distributed throughout all the communes of Ibague. Its dispersion has probably been favored by this region's environmental and social conditions. We recommend annual monitoring of these vectors populations and molecular characterization of the found arboviruses. Ascertaining this mosquito's distribution throughout the city will enable focusing entomological control strategies and preventing future arbovirus outbreaks.

La vigilancia entomológica de vectores de arbovirus es un método eficiente para determinar su densidad, variación estacional y distribución espacio-temporal, y además, permite la detección temprana de agentes patógenos, para actuar de manera oportuna y evitar posibles brotes epidémicos que pongan en riesgo la salud pública ^1^^,^^2^.

Uno de los vectores que ha generado más preocupación en los últimos años, dada su rápida dispersión y fácil adaptabilidad, es el "mosquito tigre asiático" *Aedes albopictus*^3^^,^^4^. Originario del sureste asiático, es considerado actualmente como el mosquito más invasivo del mundo y es catalogado como uno de los vectores con mayor dispersión geográfica en las últimas dos décadas ^5^. Este insecto es transmisor de arbovirus como el del dengue (DENV), el chikungunya (CHIKV), el Zika (ZIKV) y la fiebre amarilla (YFV) ^6^^-^^8^, virus que generan epidemias de enfermedades y pueden afectar gravemente la salud pública.

La introducción de este vector en el continente americano se dio en los años ochenta debido al auge del comercio de llantas usadas que sirven como transporte pasivo para los huevos, los cuales pueden sobrevivir largos periodos en total ausencia de agua. Sus formas inmaduras se detectaron por primera vez en Houston (Texas) en agosto de 1985 en llantas provenientes de Asia, y en Rio de Janeiro (Brasil) en 1986 (9,10). Los estudios de modelado de nicho ecológico han estimado que el 96,14 % del continente contiene potenciales áreas habitables para este díptero ^11^.

En Colombia, se reportó por primera vez la presencia de *A. albopictus* en 1998 en el municipio de Leticia (Amazonas) ^12^. Hasta la fecha, se ha documentado la presencia de este mosquito en 18 de los 32 departamentos del país: Amazonas, Antioquia, Arauca, Boyacá, Caldas, Casanare, Cauca, Chocó, Cundinamarca, Meta, Nariño, Norte de Santander, Putumayo, Quindío, Risaralda, Santander, Tolima y Valle del Cauca ^11^^,^^13^^,^^14^. Recientemente, se reportó la presencia de este vector en Ibagué, donde se recolectaron siete ejemplares en la comuna 9 ^15^.

En la actualidad, los planes de manejo y control vectorial se han focalizado en la fase de huevos y larvas, ya sea eliminándolos mecánicamente con el lavado regular de las albercas u otros depósitos artificiales de agua, como floreros y bebederos de animales, o con el tratamiento del agua con insecticidas para impedir el desarrollo de las larvas ^16^. Estas metodologías no son del todo efectivas con *A. albopictus,* ya que dicho mosquito se encuentra más alejado de las viviendas, tiene un amplio rango de hábitats y prefiere poner sus huevos en depósitos naturales de agua, como cáscaras de coco ^17^, bromelias ^18^, tocones de bambú, hojas de palma ^19^, huecos en los árboles ^20^ o en las rocas ^21^, axilas de hojas ^22^, conchas de caracol ^23^, brácteas de palma ^24^ y cáscaras de cacao ^23^, lugares de difícil acceso en los cuales no se pueden implementar estrategias tradicionales de control entomológico ^12^. De esta manera, la circulación y el mantenimiento de arbovirus en periodos interepidémicos se facilita en poblaciones de *A. albopictus,* ya que este se cría y desarrolla mayormente en el peridomicilio con abundante vegetación, lo que hace que su control vectorial y vigilancia entomológica sea más compleja que en las especies domiciliadas como *Aedes aegypti*^25^.

Además, la variabilidad fisiológica de *A. albopictus* le permite alimentarse de un amplio número de animales silvestres y domésticos ^24^^,^^26^^-^^28^, lo que da como resultado una rápida colonización de nuevos territorios, y le ha permitido adaptarse a diferentes ambientes, incluso con bajas temperaturas, lo que le permite tener un mayor rango de distribución en el mundo en comparación con *A. aegypti*^29^. La capacidad de resiliencia frente a los cambios de temperatura complica el diseño de predicciones y modelado de la distribución de *A. albopictus*^30^, por lo que es necesario realizar monitoreos anuales para hacer un seguimiento constante y proporcionar datos en tiempo real sobre posibles cambios de comportamiento, dispersión o preferencias de los huéspedes para la alimentación.

Ibagué cuenta con temperaturas anuales que van desde los 19 hasta los 29 °C, con una altitud promedio de 1.285 msnm, condiciones favorables para una proliferación rápida de *A. albopictus.* Sin embargo, la zona urbana de Ibagué está representada por 13 comunas con rangos altitudinales que van desde los 826 msnm (comuna 9) hasta los 1.650 msnm (comuna 13), lo que muestra la necesidad de realizar estudios que abarquen toda el área de la ciudad.

El problema del dengue en Ibagué es una preocupación significativa debido a la alta incidencia de casos registrados en los últimos años. De acuerdo con el boletín epidemiológico número 52 del Instituto Nacional de Salud del 2019, Ibagué ocupó el tercer lugar en registros de dengue con 5.127 casos ^31^ y en el 2020, según el boletín número 53, ocupó el mismo lugar con 2.764 casos ^32^. Aunque durante los años 2021 y 2022 contó con pocos casos registrados de arbovirosis en el país, se pronostica un aumento para el período 2023-2024 debido a la circulación natural de estos virus.

Dado este contexto, el objetivo principal de la presente investigación fue determinar la distribución de *A. albopictus* en las distintas comunas de Ibagué. Esta evaluación se llevó a cabo con el propósito de identificar y comprender la presencia de este mosquito como un factor de riesgo que podría contribuir a futuros brotes de arbovirosis en la zona.

## Materiales y métodos

### Área de estudio

Esta investigación se realizó en Ibagué, capital del departamento del Tolima que tiene una extensión de 1.498 km^2^, una elevación promedio de 1.285 msnm y una temperatura media de 21 °C. Ibagué está dividida políticamente en 13 comunas y su población aproximada es de 542.724 habitantes ^33^.

### Diseño de muestreo e identificación taxonómica de Aedes albopictus

Los mosquitos *A. albopictus* adultos fueron recolectados entre mayo y noviembre de 2022. Las unidades de muestreo fueron parques y zonas verdes con depósitos naturales o artificiales de agua, que representan lugares potenciales para la cría y el desarrollo del vector ^34^^,^^35^. Con el fin de conocer la distribución de este vector en Ibagué, se realizaron muestreos en todas las comunas. Hasta el 2021, únicamente se había reportado la presencia de *A. albopictus* en la comuna 9, donde se encontraron 7 ejemplares ^15^. Se realizaron dos salidas de campo por comuna, cada una de un día, para un total de dos días por comuna. Todas las salidas contaron con la misma intensidad de muestreo: dos horas de búsqueda -entre las 8 y las 10 de la mañana o entre las 4 y las 6 de la tarde- en las cuales los mosquitos tienen mayor actividad debido a su comportamiento alimenticio bimodal ^36^^,^^37^.

**Figura 1:**
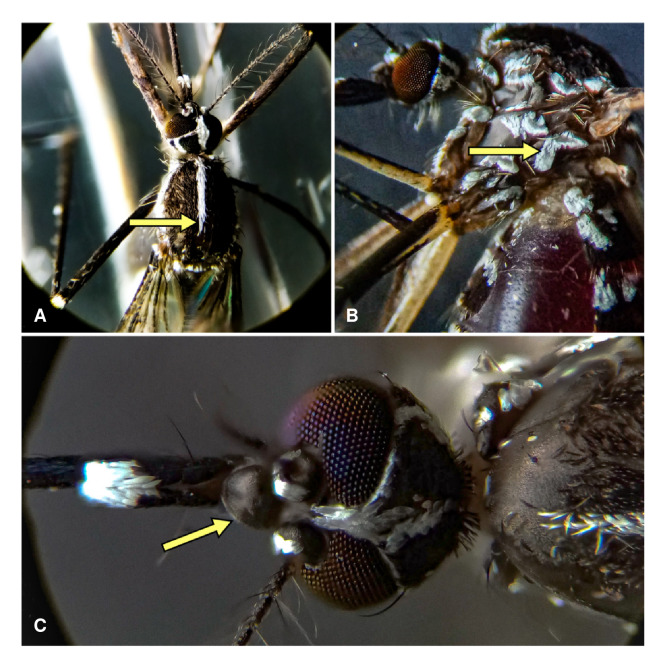
Caracteres diagnósticos de *Aedes albopictus:* (A) línea media blanca sobre el dorso del tórax; (B) patrón de escamas blancas en forma de "V" en el mesanepímero y (C) clípeo totalmente negro.

Los mosquitos adultos fueron recolectados con el acompañamiento del personal de la Secretaría de Salud Municipal de Ibagué. Se emplearon aspiradores y redes entomológicas, y los individuos recolectados se depositaron en recipientes plásticos de dos litros para su adecuado transporte. Posteriormente, fueron trasladados al Laboratorio de Investigaciones en Parasitología Tropical de la Universidad del Tolima, donde se determinaron taxonómicamente siguiendo la clave morfológica de Rueda ^38^. Los caracteres diagnósticos, como la línea media blanca sobre el dorso del tórax, el patrón de escamas blancas en forma de "V" en el mesanepímero y el clípeo sin coloración, se observaron con ayuda de un estereoscopio, como se muestra en la figura 1. Se seleccionaron únicamente los ejemplares de *A. albopictus,* que luego fueron sexados, contados y almacenados a --80 °C para análisis posteriores.

### Análisis de la abundancia de Aedes albopictus en Ibagué

Para determinar la abundancia relativa de *A. albopictus* en cada una de las comunas de la ciudad, se implementó la siguiente ecuación:




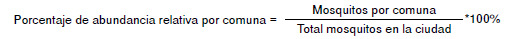




Del mismo modo, se realizó un análisis estadístico para determinar si hubo diferencia entre las medianas de los datos y así evaluar posibles cambios en la abundancia de mosquitos durante los meses evaluados.

### Análisis de datos

Se aplicó la prueba estadística Shapiro-Wilk para determinar la normalidad de los datos. Se utilizó la prueba no paramétrica de Kruskal-Wallis para evaluar las diferencias en la distribución de mosquitos por mes. Todos los análisis se llevaron a cabo con un nivel de significancia del 5 % mediante el *software* InfoStat™, versión estudiantil 2020 ^39^.

Para la elaboración del mapa de distribución, se empleó la versión educativa de ArcGIS Online™. Asimismo, se utilizó el *software* Google Earth Pro™ para obtener las coordenadas geográficas y la altitud de cada sitio de muestreo.

## Resultados

### Abundancia de Aedes albopictus en Ibagué

**Figura 2:**
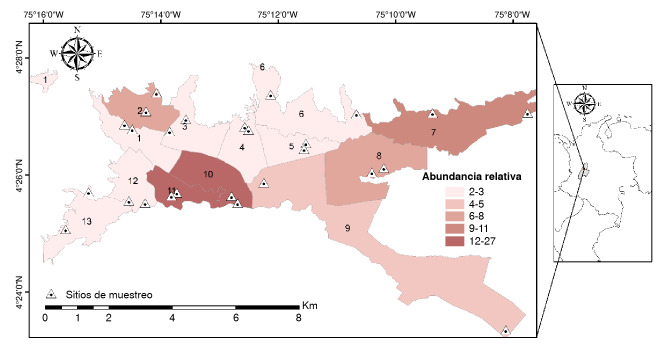
Mapa de calor de la abundancia relativa de *Aedes albopictus* por comuna y la ubicación de los sitios de muestreo

Se recolectaron 474 hembras y 234 machos para un total de 708 ejemplares del mosquito tigre asiático. Se encontró que la mayor abundancia relativa estuvo en las comunas 10 (27 %), 11 (21 %), 7 (11 %), 8 (8 %), 2 (7 %) y 9 (5 %). Las comunas 3, 4, 5, 6, 12 y 13 presentaron abundancias cercanas al 3 %. Finalmente, la comuna 1 presentó la abundancia más baja con un valor cercano al 2 %. Estos resultados reflejan que el mosquito tigre asiático está distribuido en toda la ciudad como se muestra en la figura 2.

### Abundancia de mosquitos por mes

**Figura 3:**
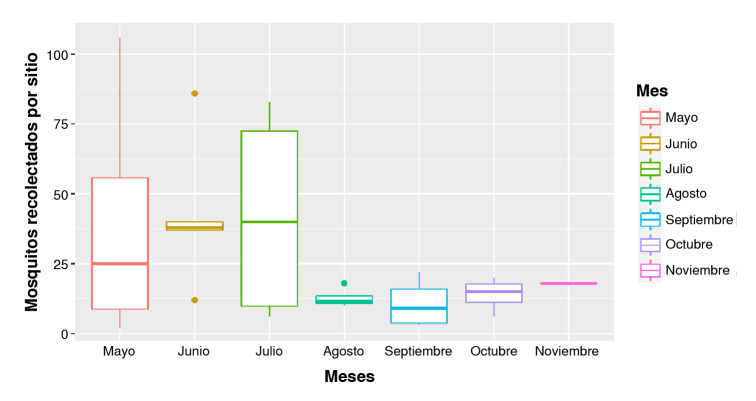
Diagrama de cajas de la cantidad de mosquitos encontrados por cada sitio de muestreo entre mayo y noviembre del 2022

El resultado de la prueba no paramétrica de Kruskall-Wallis dio un valor de 0,4807, por lo tanto, no hubo diferencia estadísticamente significativa entre la abundancia de mosquitos *A. albopictus* entre los meses de mayo y noviembre como se muestra en la figura 3. Esto podría estar relacionado con el clima variable que se presentó durante el año evaluado, ya que no hubo un patrón claro de temporada seca y lluviosa.

## Discusión

Los planes de monitoreo de mosquitos transmisores de arbovirus son estrategias necesarias para el control y la prevención de posibles brotes epidémicos que afectan gravemente la salud pública ^1^^,^^40^. La vigilancia entomológica de las poblaciones de *A. albopictus,* realizada en 2022 en Ibagué, mostró la amplia distribución de este vector en la ciudad. Se encontraron 708 ejemplares, entre los 908 y 1.374 msnm, en todas las comunas del área urbana estudiada. Aunque el primer reporte para la ciudad se realizó en el 2021 ^15^, no se conoce con certeza en qué año llegó este mosquito a la ciudad, dado que presenta múltiples formas de dispersión.

En el caso de la dispersión activa -desplazamiento por vuelo- los mosquitos pueden movilizarse entre 83 y 333 m por día, dependiendo de las condiciones climáticas de la región ^41^; y en la pasiva, los huevos pueden ser trasladados largas distancias por el comercio de llantas usadas o de plantas ornamentales como el bambú, etc. ^42^. En cuanto a individuos adultos, en Europa se ha reportado la dispersión pasiva de *A. albopictus* en vehículos terrestres que viajan desde Italia hacia Alemania, cruzando los Alpes suizos ^43^. Esta situación podría presentarse en Ibagué, donde el sistema de transporte público viaja diariamente de extremo a extremo de la ciudad.

**Figura 4:**
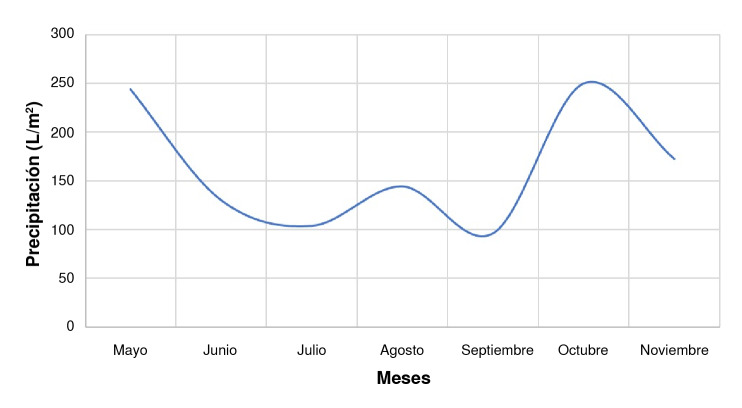
Patrón de lluvias de Ibagué de mayo a noviembre del 2022

El clima de Ibagué se distingue por su patrón bimodal de precipitaciones, que evidencia dos períodos notables de lluvia (figura 4). Los niveles de precipitación alcanzan sus valores máximos en los meses de abril y mayo, y nuevamente en el período de octubre a noviembre ^44^. Esto eleva los niveles de humedad y aumenta la cantidad de depósitos naturales de agua, preferidos por *A. albopictus,* el cual establece sus criaderos principalmente en zonas con abundante cobertura vegetal ^9^^,^^12^. La temporada seca entre los meses de julio y agosto proporciona un aumento en el promedio de la temperatura ambiental, lo que acelera el desarrollo de colonias naturales de estos insectos, pues a mayor temperatura, más rápido completa su ciclo de vida ^45^^-^^49^.

Este patrón bimodal de aumento de los niveles de precipitación seguido de un aumento en la temperatura es un factor que favorece los criaderos de mosquitos y la circulación de virus. Gutiérrez y colaboradores ^6^, encontraron que, a mayores temperaturas ambientales, se incrementa la tasa de replicación y transmisión del virus del Zika en poblaciones de *A. albopictus.*

Hasta el año 2022 se pensaba que *A. albopictus* establecía sus criaderos estrictamente en lugares peridomiciliarios con alta densidad de cobertura vegetal. Sin embargo, en el 2023, en un estudio realizado en La Habana (Cuba), Marquetti y colaboradores demostraron que cada año son más los sitios de cría que se encuentran al interior de las viviendas. Esto sugiere el inicio de domiciliación de esta especie y pone en alerta a los programas de control de vectores ^50^. De esta manera, *A. albopictus* se convierte en un vector eficiente, puente de enfermedades zoonóticas, dada su facilidad para moverse entre ambientes urbanos y selváticos, e interactuar constantemente con la diversidad viral de la fauna silvestre ^24^^,^^51^.

La temperatura de Ibagué es un factor crucial para el desarrollo de los mosquitos, su promedio anual es de 21 °C, con máximos de 29 °C y mínimos de 19 °C ^52^, lo que permite que el ciclo de vida completo de *A. albopictus* -desde la oviposición hasta la emergencia del adulto- sea de entre 10 y 17 días, dependiendo de la humedad relativa ^45^. Asimismo, se ha reportado que entre los 20 y 25°C es la temperatura a la cual las hembras adultas tienen mayor longevidad, lo que aumenta su probabilidad de picar, transmitir virus y reproducirse ^53^.

Dada la capacidad de *A. albopictus* de madurar huevos sin requerir alimentación previa con sangre ^54^, y de ovipositar entre 32 y 80 huevos por ciclo gonotrófico ^45^, una hembra en las condiciones climáticas de Ibagué estaría en capacidad de producir, aproximadamente, 280 huevos por mes, ya que cada ciclo gonotrófico tiene una duración promedio de tres días. En condiciones peridomiciliarias *A. albopictus* tiene una media de vida de 30 días, tiempo en el cual podría desarrollar cinco ciclos gonotróficos ^37^^,^^55^. La duración del ciclo gonotrófico es un factor importante: se sabe que *A. aegypti*tarda cerca de cuatro días en completarlo y *A. albopictus,* tres días. Al presentar este último un ciclo más corto, el número de picaduras sobre el huésped aumenta, lo que podría generar un incremento en la capacidad vectorial ^55^^-^^57^.

En cuanto a la situación socioeconómica, el panorama de Ibagué es complejo. Desde agosto del 2021 se han registrado 14 nuevos asentamientos humanos de origen informal o también conocidos como "invasiones", resultado de la crisis económica generada por la pandemia del COVID-19 que dejó a miles de personas en condición de vulnerabilidad ^58^. En el presente estudio se realizaron muestreos en tres de estos asentamientos informales: "Ecoparaíso", "Nueva Jerusalén" y "Villa Resistencia", donde se encontraron 88 mosquitos de la especie *A. albopictus,* en lugares con abundante vegetación, pero con presencia de basuras por el funcionamiento deficiente de los servicios básicos de recolección de residuos sólidos. Estos resultados concuerdan con los hallazgos de Thongsripong y colaboradores, quienes encontraron una asociación entre la alta actividad de oviposición de *A. albopictus* y zonas con asentamientos familiares con ingresos económicos por debajo del promedio en el sur de Estados Unidos ^59^.

Los asentamientos informales carecen de servicios de acueducto y alcantarillado, por lo que sus habitantes no tienen acceso a agua de calidad. Como consecuencia, los residentes se ven obligados a obtener agua directamente de ríos o quebradas, almacenándola en contenedores improvisados como baldes o barriles que se ubican alrededor de sus viviendas. Lamentablemente, esta situación crea un entorno propicio para la reproducción de mosquitos, ya que los depósitos artificiales como llantas usadas, macetas, contenedores de almacenamiento, frascos y latas se han identificado como sitios comunes de cría para estos insectos ^9^^,^^60^^,^^61^. Estudios previos han resaltado el papel de los tanques descartados y las llantas usadas como los sitios más propensos a convertirse en criaderos, representan el 37 y el 26 % de los sitios donde fueron encontrados insectos en estado larval. Este fenómeno puede atribuirse en parte a la escasa exposición a la luz y a los niveles óptimos de humedad relativa que se encuentran dentro de estos recipientes ^62^, de manera que el conocer los criaderos de mosquitos con mayores índices larvarios permitirá focalizar las estrategias de control entomológico. Los elementos previamente mencionados revelan cómo las condiciones tanto ambientales como sociales de Ibagué crean un entorno propicio para la multiplicación de insectos vectores de arbovirus en estas comunidades.

Los estudios realizados en Estados Unidos y Brasil sobre el papel del mosquito tigre asiático en el desplazamiento de otras especies en lugares peridomiciliarios, han demostrado que *A. albopictus* es un competidor larvario superior que *A. aegypti,* porque este último solo creció cuando la densidad larvaria fue baja y la cantidad de alimento alta, mientras que *A. albopictus* logró desarrollarse hasta su forma adulta con pocas cantidades de alimento y alta densidad larvaria, probablemente porque aprovecha mejor los recursos de hojarasca propios de los depósitos naturales de agua, lo que da como resultado un desplazamiento de *A. aegypti*^63^^,^^64^.

En el caso de Ibagué, existen 218 parques con un área de 342.056 m^2^ correspondientes a zonas verdes y 273.804 m^2^ a separadores ^65^. Esto brinda las condiciones óptimas para el desarrollo y actividad de picadura de *A. albopictus,* que se puede encontrar en zonas urbanas, semiurbanas y rurales ^66^. En el presente estudio se realizaron muestreos en nueve zonas públicas verdes, categorizadas como: cerros, parques, jardines botánicos o plazoletas. Se encontró la presencia de *A. albopictus* en todos estos lugares muestreados, lo que refleja el potencial riesgo de transmisión de arbovirus por este vector en la ciudad, ya que se encuentra establecido en zonas altamente transitadas por población humana.

Los estudios realizados por Klowden y Chambers demostraron que la capacidad de adaptación de *A. albopictus* se debe a que su período pupal es más extenso que el de *A. aegypti.* Esta prolongación en dicha etapa del ciclo de vida ayuda a la acumulación de proteínas, lípidos y azúcares, lo que le brinda mejor suministro de nutrientes a la forma adulta. La mayor acumulación de reservas energéticas confiere a *A. albopictus* dos notables ventajas: por un lado, prolonga su supervivencia en comparación con *A. aegypti* en situaciones de inanición; y, por otro, permite la producción de huevos sin la obligación de un consumo sanguíneo previo. En condiciones de laboratorio, se ha documentado una tasa de autogenia del 5 % en individuos que fueron exclusivamente alimentados con soluciones azucaradas y lograron desarrollar huevos ^54^. Esto explica por qué, incluso en lugares donde el recurso proteico de sangre no es constante, el mosquito tigre asiático sigue proliferando día tras día.

La diapausa en los mosquitos es un proceso fisiológico genéticamente programado, en el cual los embriones entran en latencia cuando las condiciones ambientales son desfavorables, lo que aumenta la probabilidad de superar alteraciones fuertes de su entorno, como las que actualmente ocurren con el cambio climático ^67^. La diapausa como estrategia de supervivencia se ha reportado en *A. albopictus,* lo que explica la distribución global de este vector ^68^. Investigaciones han mostrado que, como respuesta a las condiciones hostiles, los mosquitos aumentan la expresión de genes asociados a la diapausa como: *cathepsin, idgf4*y *pepck,* que podrían utilizarse como blancos moleculares para futuras estrategias de control entomológico ^69^.

Se puede concluir que *A. albopictus* está distribuido en toda la ciudad de Ibagué y en el presente estudio se obtuvo el primer mapa de distribución de este vector en la ciudad. Las comunas con mayor abundancia de este vector fueron la 10, 11, 7, 8 y 2, dadas las características ambientales y sociales del área. Esto refleja un alto potencial de transmisión de arbovirus a la población humana, ya que, sumado a esto, también se ha reportado la circulación del virus del dengue y del chikungunya en poblaciones de mosquitos *A. aegypti*^15^.

Hasta la fecha este es el primer levantamiento entomológico para *A. albopictus* en toda la ciudad, y como lo muestra este trabajo, su distribución ha sido favorecida por las características sociales y ambientales de la ciudad, lo que contribuye a su proliferación y, por ende, aumenta el riesgo de una alta incidencia de arbovirosis en la región. Se recomienda llevar a cabo una vigilancia entomológica anual para hacer seguimiento a las poblaciones de la especie, ejecutar bioensayos para evaluar resistencia y sensibilidad a insecticidas y realizar una detección molecular de los posibles arbovirus presentes en este vector.

Los resultados presentados aquí abren la puerta a futuras investigaciones que propongan evaluar la relación entre niveles de precipitación y temperatura de las temporadas secas y lluviosas con respecto a la abundancia de *A. albopictus* en la ciudad. Ahora se sabe que este mosquito puede hallarse en todas las comunas y que la diferencia entre la cantidad de individuos encontrados depende de las características particulares de cada localidad en una escala temporal.

El conocer la distribución completa de este vector en la ciudad sirve para focalizar y promover el desarrollo de nuevas estrategias de control entomológico, pues las metodologías implementadas actualmente se han centrado en *A. aegypti,* y debe prestarse igual atención a *A. albopictus,* un vector de arbovirus, poco conocido, que ya se estableció en Ibagué y que es de alto riesgo para la salud pública.

Por último, se sugiere llevar a cabo investigaciones adicionales que analicen los índices de las formas inmaduras del vector tanto en criaderos naturales como en artificiales. Esta información será fundamental para el desarrollo de estrategias más efectivas de control entomológico, diseñadas específicamente en función de las preferencias de sitios de cría de los mosquitos.
